# Use of aspirin in the prevention of colorectal cancer through TIGIT‐CD155 pathway

**DOI:** 10.1111/jcmm.14332

**Published:** 2019-05-14

**Authors:** Bin Ma, Xiangguo Duan, Qiunan Zhou, Juanxi Liu, Xiaojuan Yang, Dong Zhang, Shaoqi Yang, Yong Du, Hai Li, Chunxia Su

**Affiliations:** ^1^ Department of Laboratory Medicine College of Clinical Medicine, Ningxia Medical University Yinchuan China; ^2^ Department of Oncology Surgery The First People's Hospital of Yinchuan Yinchuan China; ^3^ Department of Laboratory Surgery General Hospital of Ningxia Medical University Yinchuan China; ^4^ Department of Colorectal Surgery General Hospital of Ningxia Medical University Yinchuan China; ^5^ Department of Gastroenterology General Hospital of Ningxia Medical University Yinchuan China; ^6^ Department of Pathogen Biology and Immunology, School of Basic Medical Science Ningxia Medical University Yinchuan China

**Keywords:** aspirin, CD155, CD226, cell proliferation, colorectal cancer, TIGIT

## Abstract

Colorectal cancer (CRC) is one of the most widespread malignant cancers, with a high incidence and mortality all over the world. Aspirin (ASA) otherwise known as acetylsalicylic acid, is a non‐steroidal anti‐inflammatory drug that has shown promising results in the prevention of chronic diseases, including several cancers. In previous studies, aspirin has been shown to reduce the incidence of CRC. Immune checkpoint blockade of T cell Ig and ITIM domain receptor (TIGIT) alone or combined with other immune checkpoint blockades moleculars has gained impressive results in the treatment of the melanoma and glioblastoma. Here, we found that TIGIT and Poliovirus receptor (PVR, CD155) are expressed in tumour cells; the TIGIT and CD155 protein expression in cancer tissue has been found to be significantly higher than that in the precancerous tissue. T cell Ig and ITIM domain receptor and CD226 were expressed in the lymphocytes near the tumour tissue and the adjacent tissues. Aspirin has been found to inhibit cancer cell viability and promote CRC cell apoptosis.Similarly, aspirin has also been found to increase pro‐apoptotic protein Bax's expression. We found that the expression of TIGIT decreased with an increase in the concentration of aspirin and that the suppression of TIGIT can affect the effect of aspirin on cell proliferation. In this paper, we found that aspirin attenuates cancer cell proliferation and induces CRC cells apoptosis by down‐regulating the expression of TIGIT, which provides new evidence for the application of aspirin in cancer treatment.

## 1 INTRODUCTION

Colorectal cancer (CRC) is one of the most widespread malignant cancers, with a very high incidence and mortality rate. There are numerous factors that contribute to CRC, which include eating habits, smoking and chronic inflammatory bowel disease. However, in the majority of the cases, colorectal cancer is diagnosed only in the advanced stage. Despite the advancement in the treatment methodologies, the prognosis and the 5‐year survival rate of colorectal cancer are still very low. The most important thing is early detection of the disease and its prevention. In this paper, we found that aspirin attenuates cancer cell proliferation and induces CRC cell apoptosis by down‐regulating the expression of T cell Ig and ITIM domain receptor (TIGIT), which provides new evidence for the application of aspirin in cancer treatment in clinics.

Aspirin is a non‐steroidal anti‐inflammatory drug used in the treatment of cardiovascular diseases. In recent decades, there have been many studies on aspirin and its effectiveness in the treatment of colorectal cancer. In pre‐clinical studies, aspirin has been found to reduce the incidence of CRC^1^ and it is used as secondary prevention in patients with CRC.^2^ Aspirin can inhibit cell proliferation and pro‐apoptosis in several tumour‐derived cell lines, the mechanism of which is through the inhibition of the Wnt/β‐catenin pathway,^3^ vascular endothelial growth factor (EGFR) activation,^4^, ^5^, ^6^ NF‐κB^7^ and COX‐2 expression.^8^

In the immune system, there are two types of immune responses, the innate immune response and the adaptive immune response. Memory T cell mediated cellular immune response and B cell mediated humoural immune response are the characteristics of the adaptive immune response. The tumour microenvironment is the first location in which the tumour cells and the host immune system interact. T cell activation depends on dual signal and cytokine action. The first signal of T cell activation comes from the specific binding of T cell Receptor (TCR) to the antigen and the second signal from co‐stimulatory molecules. Co‐inhibitory molecules, such as PD‐1, TIM‐3, LAG‐3 and TIGIT, play a role in the activation of T cells, of which TIGIT is found to be expressed on activated T cells and NK. Immunosuppression is one of the causes of tumour escape in the tumour environment. Few studies have explored the relationship between aspirin and TIGIT.Therefore, in this paper, we examine the relationship between TIGIT and the tumours in CRC.

Poliovirus receptor (PVR, CD155), a member of the nectin‐like family of protein, has been recently seen as a promising target in immunotherapy that enhances antitumour responses. Poliovirus receptor is dramatically overexpressed in several human malignant cancers, however, its expression is low or absent in most healthy tissues.^9^, ^10^, ^11^, ^12^, ^13^ CD155 has an immunoregulatory potential. It can interact with CD226, TIGIT and CD96 exerting co‐stimulatory and co‐inhibitory signals.^14^ However, the balance was disturbed in the tumour microenvironment which induces the tumour cells to escape from the immune surveillance resulting in tumour formation.

## 2 MATERIALS AND METHODS

### 2.1 Drugs

Aspirin was purchased from Sigma (American) and dissolved in anhydrous ethanol just before use.

### 2.2 Cell culture

The colorectal cancer cell line HT‐29 cells were purchased from American Type Culture Collection and were cultured in RPMI 1640 medium (Hyclone) containing 10% foetal bovine serum (FBS, PANBiotech).

### 2.3 Immunohistochemistry analysis

A 4‐µm thick section of CRC and paracancerous tissues were prepared. The tissues were heated at 64°C for 2 hours, after which the tissue sections were subjected to immunohistochemical staining. Subsequently, the sections were deparaffinized in xylene and dehydrated in a graded alcohol series (ethyl alcohol, 95% alcohol, 90% alcohol, 80% alcohol, 75% alcohol and 50% alcohol). The endogenous peroxidase activity was blocked for 20 minutes using 3% H_2_O_2_, following which the antigen was retrieved using the ethylenediaminetetraacetic acid antigen repair solution (pH 9.5) in a high‐pressure steam boiler for 3 minutes. After non‐specific binding was blocked using normal goat serum at 37°C for 1 hour, the sections were incubated overnight with anti‐TIGIT, anti‐CD226 and anti‐CD155 antibodies(Abcam) in PBS at 4°C. After incubation with the primary antibody, the sections were treated with secondary antibodies at room temperature for 30 minutes, stained with diaminobenzidine for 1.5 minutes and then counterstained with hematoxylin for 4 minutes.

### 2.4 Cell viability

HT‐29 cells were seeded in 96‐well microplates (1.5 × 10^4^ cells per well) in a total volume of 200 µL and cultured with various doses of aspirin for 24, 48 and 72 hours. Cell Counting Kit (CCK 8) solution (Shanghai BestBio, China) was added (10 µL per well) to the cells and the cells were incubated for another 2 hours at 37°C. The optical density was measured at an absorbance of 450 nm.

### 2.5 Migration assay

Scratch assay was performed to analyse cell migration in vitro; HT‐29 cells were grown in six well plates and allowed to adhere for 24 hours. A wound was generated when the cells reached 90% confluence by scratching the surface of the plates with a 10 µL pipette tip. The cells were then washed twice with PBS to remove the detached cells, incubated with aspirin (2, 4, 8 mmol/L) for 72 hours and photographed with a microscope.

### 2.6 Apoptosis detection

Apoptosis was detected using flow cytometry. An Annexin V‐FITC/Propidium iodide (PI) double stain assay (KeyGen Biotech Co. Ltd., Nanjing, China) was performed following the manufacturer's protocol. Following this, the untreated and treated cells were washed with PBS buffer, gently suspended in the buffer and incubated with Annexin V‐FITC and PI (Propidium iodide) for 15 minutes in the dark. Flow cytometry was performed with the BD Accuri C6 software.

### 2.7 Western blot analysis

Both treated and untreated cells were washed twice with ice‐cold PBS and extracted on ice with cell lysis buffer (KeyGen Biotech Co. Ltd., Nanjing, China). The concentration of the protein was quantified using the BCA Protein Assay Kit (KeyGen Biotech Co. Ltd., Nanjing, China). Thirty micrograms of total protein was electrophoresed through 10% and 12% SDS PAGE and was then transferred to membranes (Millipore, USA). The membranes were blocked in 5% fat‐free milk for two hours then incubated with primary antibodies at 4°C overnight. Secondary antibodies were labelled with Horseradish Peroxidase (Abbkine Scientific, China) and the signals were detected using the ECL Kit (Advansta, UK). Subsequently, the images were analysed using the ImageJ 6.0 software. A glyceraldehyde‐3‐phosphate dehydrogenase (GAPDH) antibody was used as the control for whole‐cell lysates.

### 2.8 Statistical analysis

Continuous variables are expressed as the median with the range or as the mean ± SD. Statistical comparisons were made using ANOVA. All statistical calculations were performed with spss23.0 and a two‐sided *P*‐value < 0.05 indicated statistical significance.

## 3 RESULTS

### 3.1 Expression of TIGIT/CD226/CD155 in colorectal tissues

**Figure 1:**
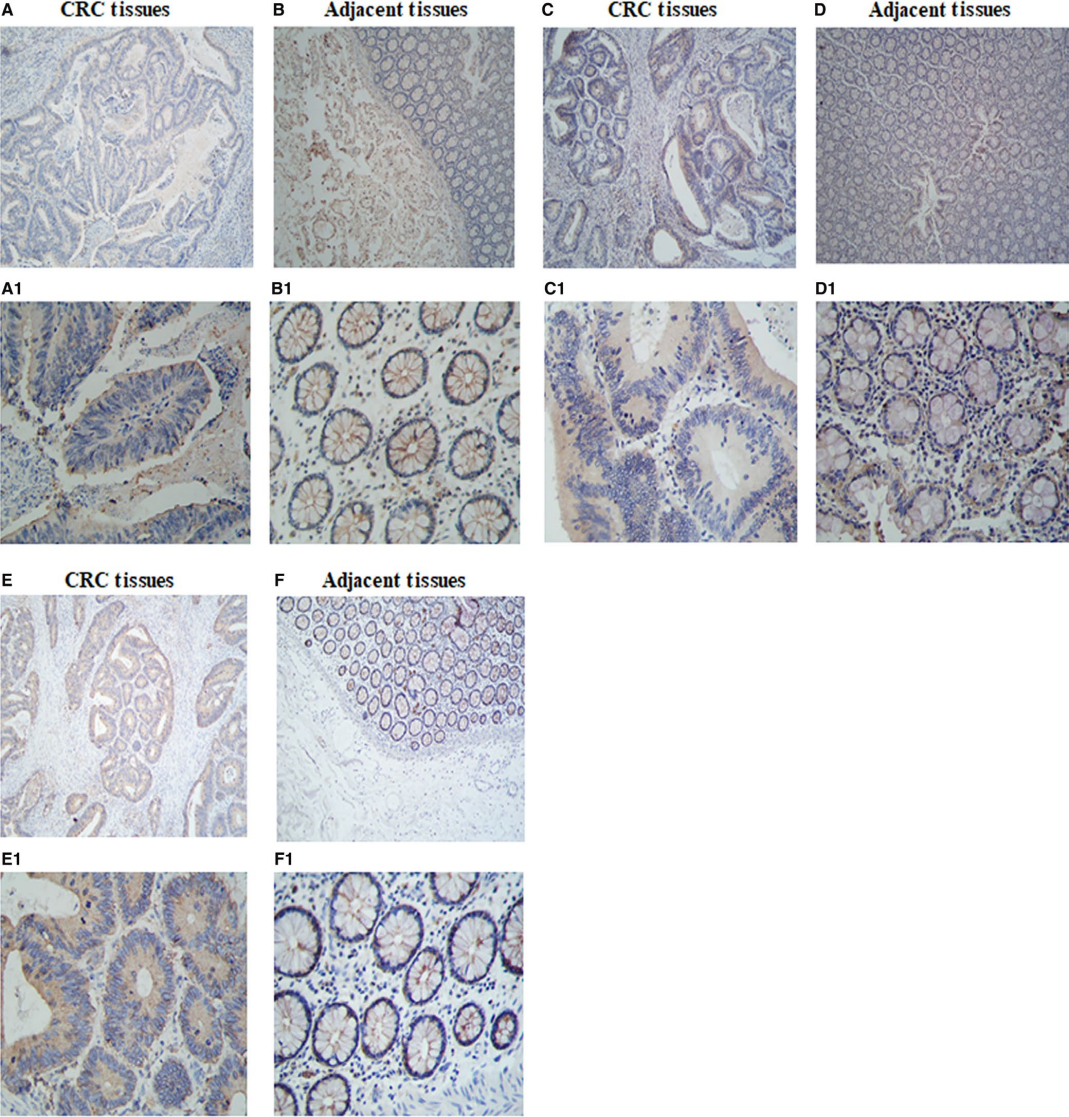
TIGIT/CD226/CD155 expression in colorectal cancer and adjacent tissues. TIGIT and CD155 are expressed in malignant epithelial cells in colorectal carcinoma. CD226 is not expressed in colorectal cancer tissues. (A,A1) Colon adenocarcinoma shows immunoreactivity with polyclonal antibody TIGIT (X40, X400). (B,B1) Adjacent section stained with an irrelevant polyclonal antibody TIGIT (X40, X400). (C,C1) Colon adenocarcinoma shows immunoreactivity with polyclonal antibody CD226 (X40, X400). (D,D1) Adjacent section stained with an irrelevant polyclonal antibody CD226 (X40, X400). (E,E1) Colon adenocarcinoma shows immunoreactivity with polyclonal antibody CD155(X40, X400). (F,F1) Adjacent section stained with an irrelevant polyclonal antibody CD155(X40,X400). TIGIT, T cell Ig and ITIM domain receptor

To determine if the cells expressed TIGIT/CD226/CD155, immunohistochemical staining was performed on 4‐µm thick section of paraffin‐embedded tissue with polyclonal antibodies(ab61790, ab136311, ab123252). T cell Ig and ITIM domain receptor was expressed mainly in the plasma membrane of cancer cells (Figure 1A,A1). CD226 was not expressed in CRC cells (Figure 1C,C1). CD155 was abundant and expressed mainly in the plasma membrane and cytoplasm of cancer cells (Figure 1E,E1).

### 3.2 Expression of TIGIT/CD226/CD155 on lymphocytes in the CRC cells and adjacent tissues

**Figure 2:**
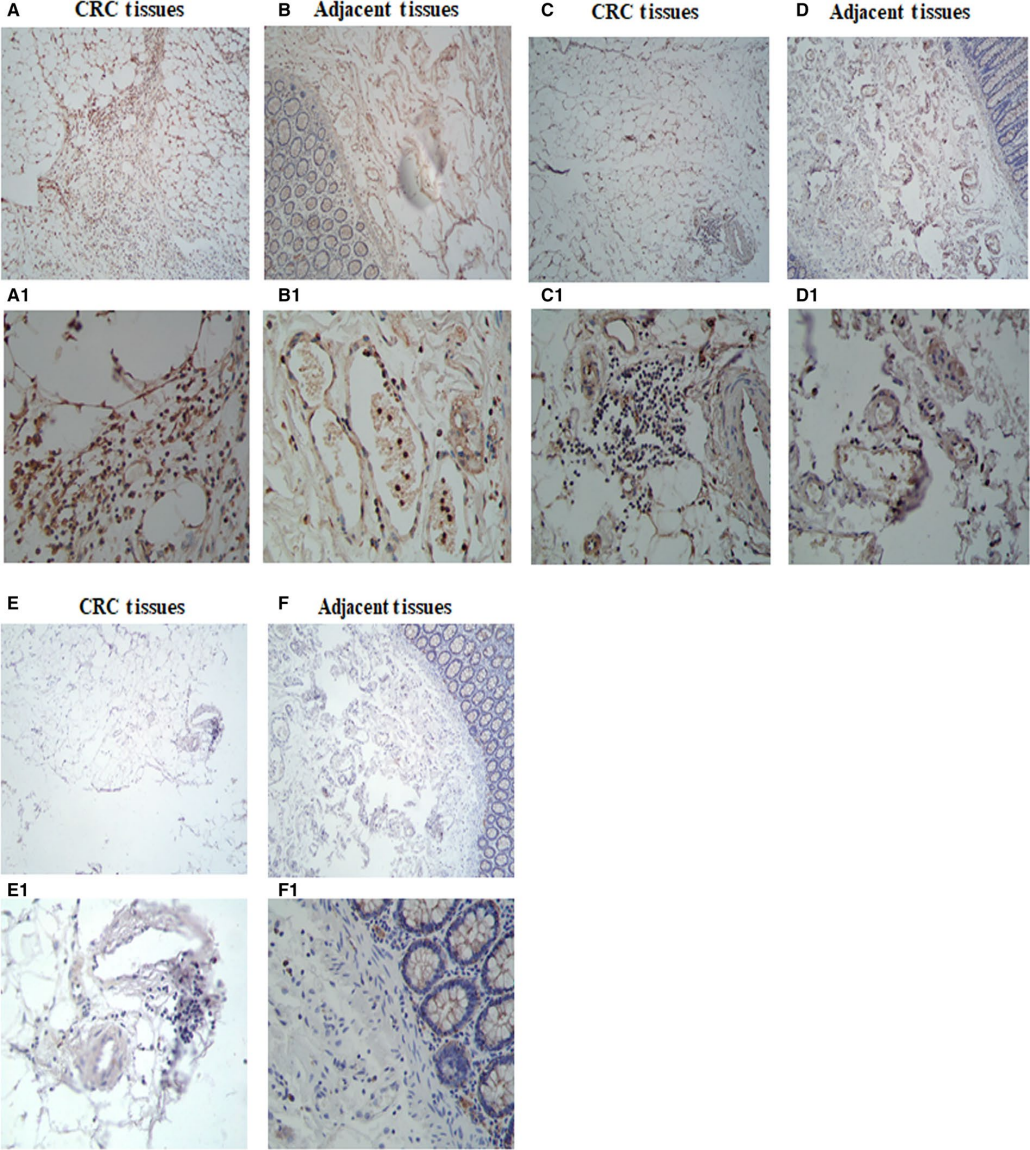
TIGIT/CD226/CD155 expressions on lymphocyte in colorectal cancer and adjacent tissues. TIGIT and CD226 are expressed in lymphocyte cells in colorectal carcinoma. CD155 is not expressed in lymphocyte. (A,A1) Colon adenocarcinoma shows immunoreactivity with polyclonal antibody TIGIT (X40, X400). (B,B1) Adjacent section stained with an irrelevant polyclonal antibody TIGIT (X40, X400). (C,C1) Colon adenocarcinoma shows immunoreactivity with polyclonal antibody CD226 (X40, X400). (D,D1) Adjacent section stained with an irrelevant polyclonal antibody CD226 (X40, X400). (E,E1) Colon adenocarcinoma shows immunoreactivity with polyclonal antibody CD155(X40, X400). (F,F1) Adjacent section stained with an irrelevant polyclonal antibody CD155 (X40, X400). TIGIT, T cell Ig and ITIM domain receptor

To observe the expression of TIGIT, CD226 and CD155 on lymphocytes in the CRC cells and adjacent tissues, immunohistochemical staining was performed on 4‐µm thick sections of paraffin‐embedded tissues with polyclonal antibodies (ab61790, ab136311, ab123252). In our experiment, we found that TIGIT was highly expressed in the lymphocytes of the CRC tissues than in the adjacent tissues lymphocytes. T cell Ig and ITIM domain receptor was expressed mainly in the plasma membrane of the lymphocyte cells (Figure 2A,A1,B,B1). CD226 was expressed mainly in the plasma membrane of the lymphocyte cells (Figure 2C,C1,D,D1). In our results, CD155 was not expressed in the lymphocytes (Figure 2E,E1,F,F1).

### 3.3 Role of aspirin in the inhibition of cell viability in CRC cells

**Figure 3:**
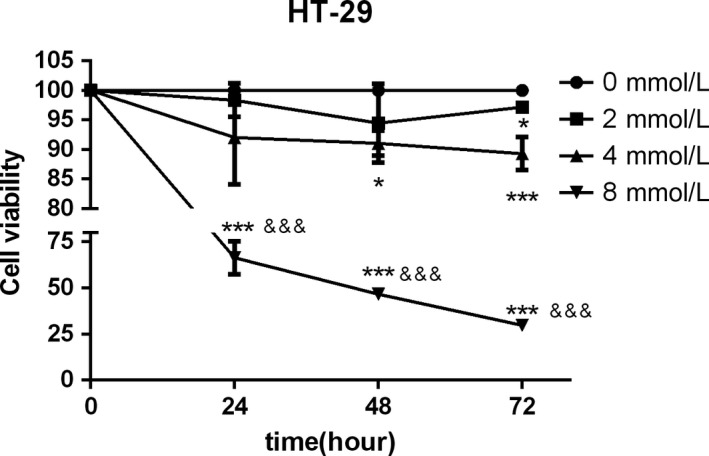
Aspirin inhibits the viability of colorectal cancer cell. HT‐29 cells were treated with increasing concentrations of ASA (from 2 to 8 mmol/L). After 24, 48 and 72 h of incubation, cell viability was measured using CCK‐8 assay. (*Significantly different from control; *P < 0.05; **P < 0.01; ***P < 0.001; ^&^Significantly different from control 0 h, ^&^ *P* < 0.05; ^&&^ *P* < 0.001; ^&&&^ *P* < 0.0001

To evaluate the potential effect of aspirin on cell proliferation in CRC cells, we first conducted the CCK8 assay using different doses of aspirin in HT‐29 cells. Concentrations between 2 and 8 mmol/L were tested at 24, 48 and 72 hours (Figure 3). CCK8 assays showed that the 50% inhibitory concentration (IC50) of 24 hours aspirin for HT‐29 was 9.0 mmol/L. Aspirin, at a concentration of 8 mmol/L, showed a significant reduction in the cell viability in HT‐29 cells. We observed that when the appropriate concentration of aspirin was reached, the inhibition of cell activity was both time‐ and concentration‐dependent.

### 3.4 Role of aspirin in the inhibition of HT‐29 cell migration in CRC cells

**Figure 4:**
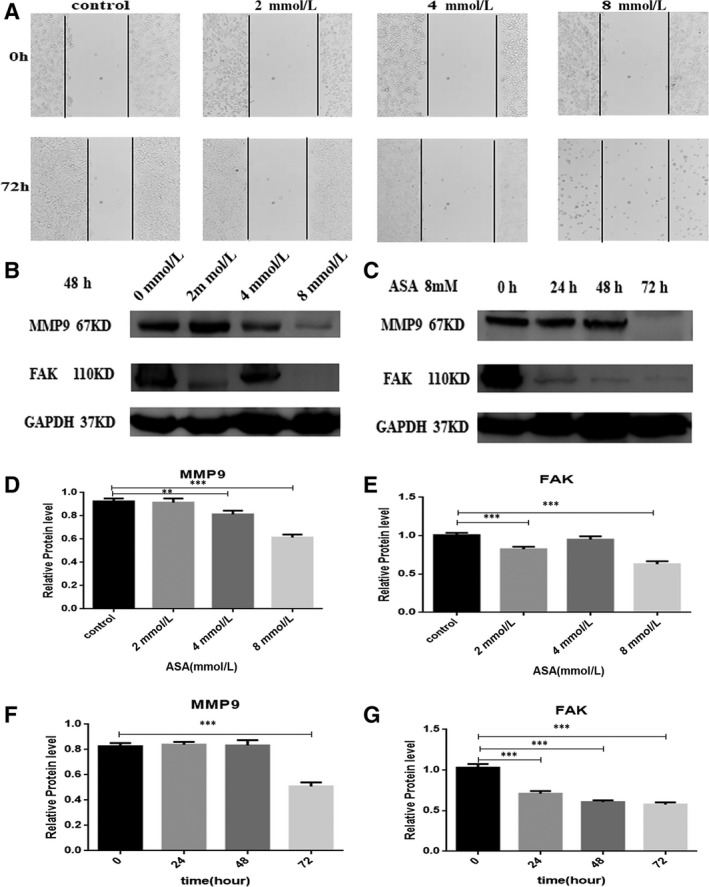
Aspirin inhibits the migration of colorectal cancer cell. A, HT‐29 cells were treated with increasing concentrations of ASA (from 2 to 8 mmol/L). After 72 h of incubation, the distance was photographed. (B‐G) The expression of MMP‐9 and FAK proteins was deteced using western blotting in HT‐29 cells. Antibody against GAPDH was used as loading control.**P < 0.01, ***P < 0.001, compared to control group. All data are expressed as mean ± SD of three separate experiments

The scratch assay was carried out to analyse the role of aspirin in the inhibition of the migration of HT‐29 cells. Treatment with a aspirin was found to induce the migration of HT‐29 cell lines at 72 hours (Figure 4A). However, the data show that aspirin can inhibit the migration of the HT‐29 cells. We also explore the mechanism of aspirin inhibiting the migration ability of HT‐29 in CRC cells. Our data revealed that aspirin could inhibit the expression of Focal Adhesion Kinase (FAK) and Matrix Metalloproteinases (MMP9) with increasing time and concentration (Figure 4B,C).

### 3.5 Role of aspirin in the enhancement of the apoptotic rate of HT‐29 cells

**Figure 5:**
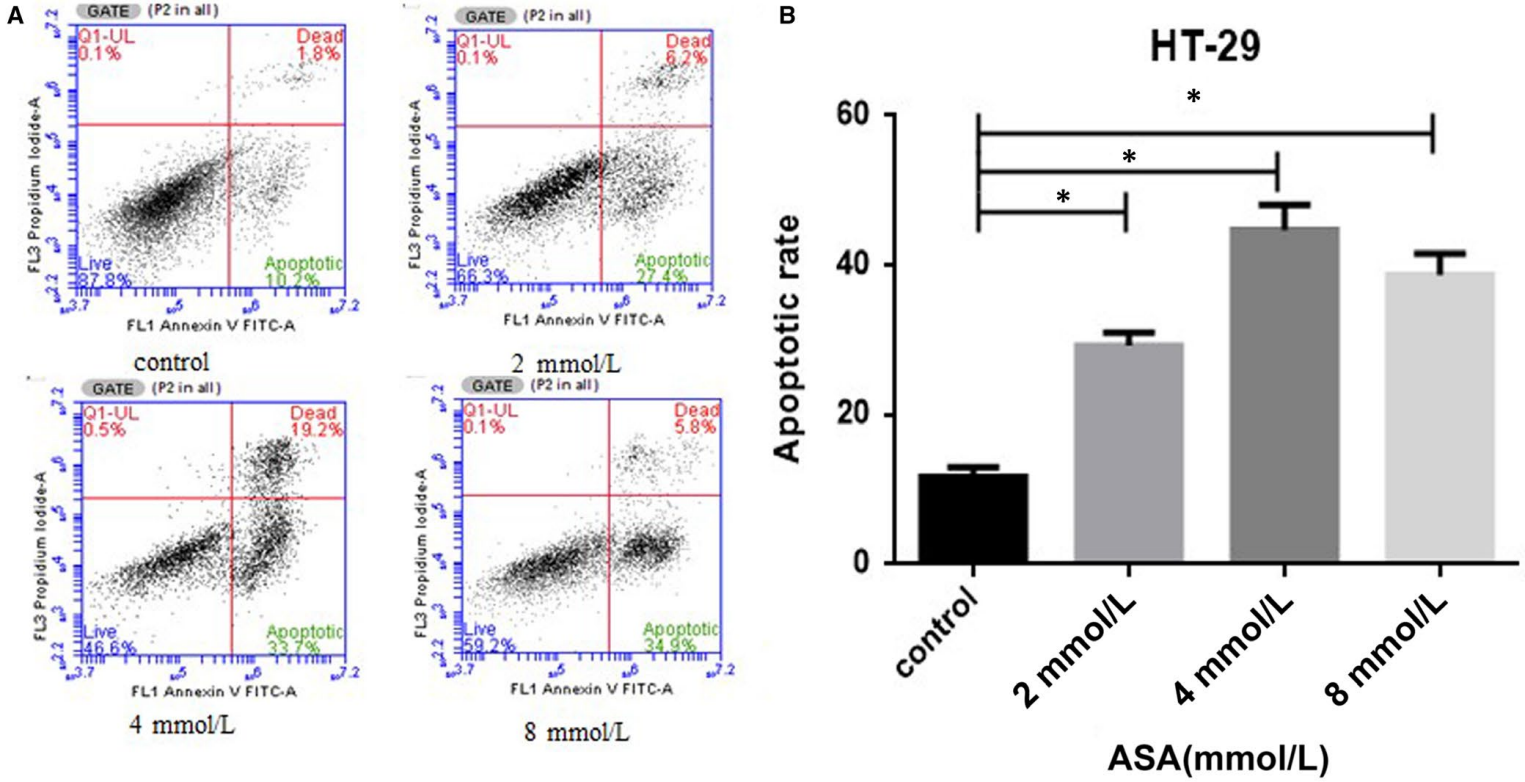
Aspirin induces the apoptotic of colorectal cancer cell. (A,B) HT‐29 cells were stained with annexin V‐FITC and PI following treatment with increasing concentrations of ASA (from 2 to 8 mmol/L) for 48 h. Data are indicative of three separate experiments.The statistical significance of the results was analysed using one way ANOVA followed by Least Significant Difference test. *P < 0.05, significantly different from control

To define the type of cell death induced by aspirin, we assessed the rate of apoptosis (cell death) of HT‐29 cells by flow cytometry using co‐staining byAnnexin V and propidium iodide. When compared with the untreated control, those treated with aspirin induced a increase in the rate of apoptosis of HT‐29 cells (Figure 5A,B).Our data show that aspirin can enhance the apoptosis of the CRC cells.

### 3.6 Effects of aspirin on anti‐apoptotic and pro‐apoptotic proteins

**Figure 6:**
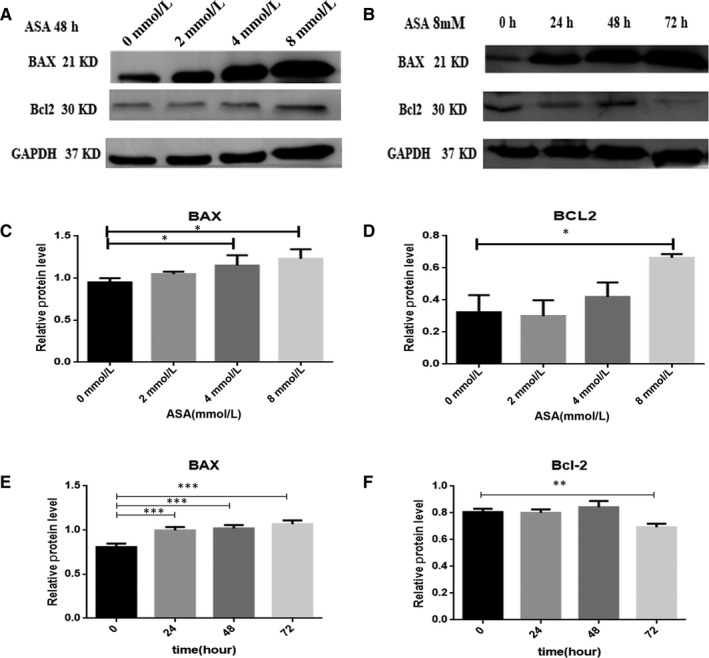
Western blot for Bax, Bcl‐2 in HT‐29 cells after treatment with ASA. A, Effects of treatment of HT‐29 cells for 48 h with IC50 concentration of ASA or different concentrations ASA(2, 4, 8 mmol/L) on for Bax and Bcl‐2 level. B, Effects of treatment of HT‐29 cells with 8mM aspirin for 24, 48 and 72 h. Antibody against GAPDH was used as loading control. *P < 0.05, **P < 0.01, ***P < 0.001, compared to control group. All data are expressed as mean ± SD of three separate experiments

To further consider the mechanism of apoptosis induced by aspirin, we investigated the protein levels of Bcl‐2 and Bax. Aspirin was found to accelerate the expression of the pro‐apoptotic protein Bax with an increase in its concentration and time (Figure 6A,B).Whereas aspirin cannot influence the expression of the anti‐apoptotic protein Bcl‐2 with an the increase in its concentration. It was found that aspirin could reduce the expression of the anti‐apoptotic protein Bcl‐2 with an increase in time at 8 mmol/L(Figure 6A,B).

### 3.7 Role of aspirin in the attenuation of the expression of TIGIT in CRC cells

**Figure 7:**
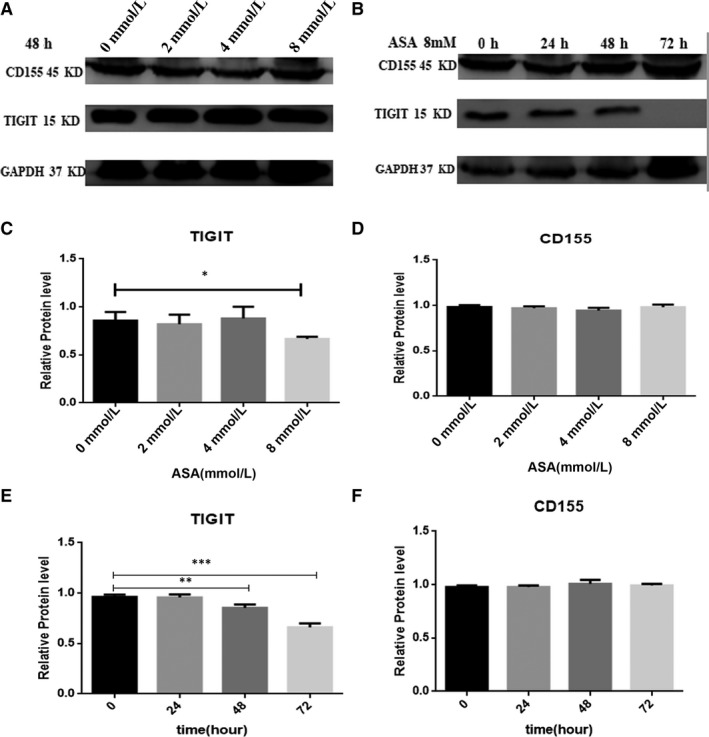
Western blot for TIGIT and CD155 in HT‐29cells after treatment with ASA. A, Effects of treatment of HT‐29 cells for 48 h with IC50 concentration of ASA or different concentrations ASA(2, 4, 8 mmol/L) on for Bax and Bcl‐2 level. B, Effects of treatment of HT‐29 cells with 8 mmol/L aspirin for 24, 48 and 72 h. Antibody against GAPDH was used as loading control. *P < 0.05, **P < 0.01, ***P < 0.001, compared to control group. All data are expressed as mean ± SD of three separate experiments. TIGIT, T cell Ig and ITIM domain receptor

To identify the effect of aspirin on the attenuation of TIGIT expression in CRC cells, we used CRC cells containing different doses of aspirin, incubated with HT‐29 cells for 48 hours. We then used 8 mmol/L aspirin incubated with HT‐29 cells for 24, 48, 72 hours to observe the influence on the attenuation of the expression of TIGIT. The results showed that aspirin inhibits the expression of TIGIT in CRC cells. However, there was no change in the expression of CD155 (Figure 7A,B).

## 4 DISCUSSION

There are several interactions between cells in the tumour microenvironment, such as cancer cells with cancer cells and cancer cells with immune cells.Thus, tumour cells and immune cells may trigger inhibitory pathways in effector T cells. Immune cells can express various receptors on its surface, l such as PD‐1, CTLA‐4, TIM‐3 and TIGIT. In these inhibitory receptors TIGIT can combine with CD155 to inhibit the T cell function.^15^ We explored the role of TIGIT in CRC as immune escape is extremely important in a tumour microenvironment.

Aspirin has been shown to directly induce antitumour effects by inhibiting the Wnt/β‐catenin pathway^3^ and NF‐κB^7^ signalling pathways, which is critical for cancer progression. In recent years, aspirin has attracted more and more attention on various chronic diseases including cancers. Recently, a team of researchers found that aspirin can be used in the treatment of Alzheimer's disease.^16^ This is a good start for researchers to discover the more potential effects of aspirin. We also want to discover the relationship between aspirin and TIGIT.

CD226 is expressed frequently by T cells and it has been shown to provide an activation signal, which is in contrast to its inhibitory effects transmitted by TIGIT engagement. Our study revealed that TIGIT can be highly expressed in the lymphocytes of tumour tissues. Simultaneously, CD155 expression has been shown to be up‐regulated in tumour cells. In this way, TIGIT can compete with CD226 for CD155 to exert an inhibitory signal. It can promote tumours to evade the immune system and cause immune escape.

In this study, we provided new evidence that TIGIT can be expressed in cancer cells and that aspirin can attenuate the TIGIT expression in CRC cells. We showed that aspirin inhibited the viability of CRC cells.We also showed that aspirin can induce cell apoptosis with an increase in its concentration. The protein level of Bax was up‐regulated in our experiment. The protein level of Bcl‐2 was down‐regulated with increasing time. We also found that, with an increase in time and concentration of aspirin, it can inhibit the migration of colorectal cells and down‐regulate the expression of migration proteins MMP9 and FAK.

In this paper, we found that aspirin attenuates cancer cell proliferation and induces CRC cell apoptosis by down‐regulating the expression of TIGIT, which provides new evidence for the application of aspirin in cancer treatment. It was also observed that aspirin repressed TIGIT expression in proteins with increasing time. These data indicated that aspirin may be a possible agent for cancer therapy by targeting TIGIT.

## CONFLICT OF INTEREST

The authors declare no conflict of interest.
